# Exploring CAR-T Cell Therapy Side Effects: Mechanisms and Management Strategies

**DOI:** 10.3390/jcm12196124

**Published:** 2023-09-22

**Authors:** Yugu Zhang, Diyuan Qin, Arthur Churchill Shou, Yanbin Liu, Yongsheng Wang, Lingyun Zhou

**Affiliations:** 1 Division of Thoracic Tumor Multimodality Treatment, Cancer Center, West China Hospital, Sichuan University, 37 GuoXue Lane, Chengdu 610041, China; yuguzhang416@gmail.com (Y.Z.); di-yuan.qin@scu.edu.cn (D.Q.); 2 Center of Infectious Diseases, West China Hospital of Sichuan University, 37 GuoXue Lane, Chengdu 610041, China; arthurshou01@163.com (A.C.S.); dr_liuyanbin@foxmail.com (Y.L.)

**Keywords:** CAR-T cell, immune-effector-cell-associated neurotoxicity syndrome, cytokine release syndrome, mechanisms, management, strategies

## Abstract

Chimeric antigen receptor (CAR) T cell therapy has revolutionized the treatment of malignancies, especially hematological tumors, but toxicities have tempered its success. The main impediments to the development of CAR-T cell therapies are the following: cytokine release syndrome (CRS), immune-effector-cell-associated neurotoxicity syndrome (ICANS), tumor lysis syndrome (TLS), and on-target/off-tumor toxicity (OTOT). This review summarizes these side effects’ underlying mechanisms and manifestations over time. It provides potential prevention and treatment according to the consensus grading, stressing the significance of establishing strategies that anticipate, reduce, and navigate the beginning of these side effects. It is essential to fully comprehend the mechanisms underlying these toxicities to create efficient treatment and preventive approaches.

## 1. Introduction

T cells can be genetically engineered to host chimeric antigen receptors (CARs), which can enable the identification and elimination of cancer cells. CARs usually consist of a single-chain variable fragment (scFv, extracellular ligand-binding domain), a spacer domain, a transmembrane region, and intracellular domains [1]. Numerous CAR-T cell treatments have demonstrated exceptional clinical success in treating hematologic malignancies [2,3] and displayed the immense promise of this ground-breaking technique for cancer immunotherapy.

Leukapheresis is a procedure used to extract T cells from the patient’s blood in the initial treatment stages. After being collected and separated, the T cells are engineered in vitro to generate a CAR to detect specific antigens. The CAR-T cells are then multiplied in a laboratory to increase their number. Prior to the CAR-T cell reinfusion, patients may undergo conditioning therapy, also referred to as lymphodepletion chemotherapy (LDC) [2,3].

**Figure 1:**
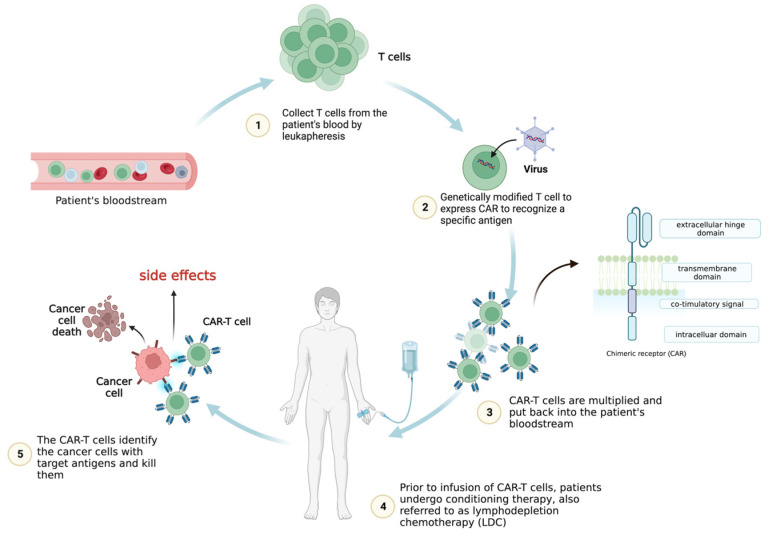
CAR-T cell therapy. The CAR-T cells are expanded before infusion into the patient’s bloodstream following lymphodepletion chemotherapy (LDC). Once in the body, the genetically modified chimeric antigen receptors (CARs) allow the T cells collected from the patient to recognize and attack cancer cells expressing specific antigens.

Radiotherapy or chemotherapy may be required to eliminate the patient’s immune cells, especially T cells that could compete against CAR-T cells for cytokines, nutrition, or other resources. The patient’s blood undergoes infusion once more with engineered CAR-T cells, which can travel to the malignant location and attack the tumor cells that express antigens recognized by the CAR. After the infusion, the effectiveness of CAR-T cell therapy and its side effects are closely monitored in the patients (Figure 1).

CAR-T cell therapy has demonstrated exceptional therapeutic effectiveness in patients with leukemia and B cell lymphoma who have relapsed or been resistant to treatment, showing high remission rates and exhibiting tremendous potential for treating additional hematological malignancies [4,5,6]. Despite these encouraging findings, therapy-related severe adverse effects are frequent and can be fatal [7].

In light of the growing adoption of CAR-T cell treatment in malignant tumor implementation, it is imperative to comprehensively elucidate the underlying pathophysiological mechanisms driving treatment-related toxicity and clinical manifestation and identify risk factors associated with its development. Such efforts are vital to mitigate potential adverse effects and optimize the therapeutic efficacy of CAR-T cell therapy in clinical settings. This review provides an in-depth overview of the various toxicities identified following CAR-T cell infusion. Additionally, we review the clinical manifestations and related management strategies according to the toxicity grading systems.

## 2. Mechanism and the Clinical Manifestations of Side Effects

### 2.1. Cytokine Release Syndrome (CRS)

The most severe prevalent toxicity in CAR-T cell therapy is an overall inflammatory reaction termed cytokine release syndrome (CRS) [8,9,10]. Tumor antigen recognition triggers CAR-T cell activation and CRS, a state of severe systemic inflammation. Following CAR-T cell injection, patients’ serum levels of cytokines, including interferon-gamma (IFN-γ), interleukin-6 (IL-6), tumor necrosis factor-alpha (TNF-α), granulocyte–macrophage colony-stimulating factor (GM-CSF), IL-2, IL-8, and IL-10, are produced by CAR-T cells and elevated, resulting in a cytokine storm. This is followed by a second inflammatory response involving antigen-presenting cells (APCs) such as dendritic cells (DCs), B cells, macrophages, and monocytes displaying the cell surface protein CD40. On activated CAR-T cells, the ligand for CD40 (CD40L) is also substantially expressed.

APC recognition favors the binding of CD40 and CD40L, which is contact-dependent, improves APCs’ antigen presentation, and stimulates the release of cytokines, including IL-1β, IL-6, and TNF-α [11,12]. Among these cytokines, IL-6 contributes to cytokine synthesis, stimulating DCs, macrophages, monocytes, and other bystander immune cells, which release additional cytokines. This phenomenon results in an enhanced inflammatory response. It creates a positive feedback loop that supports cytokine release, which may have important consequences for the onset and development of inflammatory illnesses. Robust IL-6 secretion leads to more severe CRS symptoms. The greater the IL-6 secretion, the more severe the CRS. Indeed, IL-6 is presently considered the primary contributor to toxicity in CRS [13].

**Figure 2:**
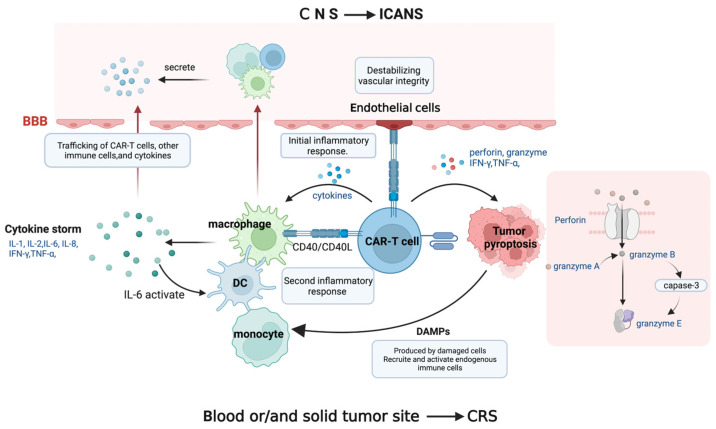
The underlying mechanisms of CRS and ICANS. CAR-T cells produce cytokine storm, which activates bystander immune cells. When CD40L on CAR-T cells interacts with CD40 on immune cells and endothelial cells, an inflammatory response occurs, with interleukin-6 (IL-6) playing a major role in the cytokine release positive feedback loop, compromising vascular integrity. The perforin produced by CAR-T cells induces tumor pyroptosis, releasing danger-associated molecular patterns (DAMPs). CAR-T cells, immune cells, and cytokines can penetrate the disrupted blood–brain barrier (BBB) and trigger inflammatory reactions in the central nervous system (CNS), resulting in neuronal injury.

Activated endothelial cells release IL-6 and endothelial permeability factors in the setting of hyperinflammation [14]. This also mediates hemodynamic dysfunction, a capillary leak, and consumptive coagulation disorders in CRS patients by destabilizing vascular integrity. Furthermore, it should be noted that endothelial cells can be activated by CD40, thereby implicating CD40/CD40L interactions that result in the upregulation of adhesion molecules and increased angiopoietin-2, resulting in organ malfunction, capillary leakage, tissue edema, and hypotension (Figure 2) [15,16].

In addition to the inflammatory response caused by immune cells, tumor cell pyroptosis during CAR-T cell therapy also causes CRS. As previously mentioned, CAR-T cells secrete perforin, granzyme, TNF-α, and IFN-γ following antigen recognition. Granzyme A and B enter the cytoplasm through the pores formed by perforin molecules. CAR T cells quickly activate caspase 3 in target cells by releasing granzyme B, which cleaves gasdermin E and causes extensive pyroptosis [17]. In the meantime, gasdermin B is activated and cleaved by granzyme A from cytotoxic lymphocytes, causing target cell pyroptosis [17,18]. As a result, components released during pyroptosis induce macrophages to promote caspase 1 for gasdermin D cleavage, which causes the amplification of cytokines and eventual CRS. After cancer cell pyroptosis, these damaged and dying cells create or release danger-associated molecular patterns (DAMPs). The endogenous immune cells, including macrophages and dendritic cells, are subsequently recruited, and activated by these DAMPs, amplifying the inflammatory response, and increasing the release of cytokines like IL-1β and IL-6. This series of events sheds light on the intricate mechanisms behind CAR-T cell therapy’s therapeutic effect [19].

The clinical manifestation of CRS occurs when a substantial number of myeloid cells or immunological cells become activated and release inflammatory cytokines [20]. The triggering factor and the activation level of immune cells determine when symptoms will start to appear and how severe CRS will be. The initial symptom is almost invariably a fever, which can occasionally reach 40.58 °C or higher; some general signs are tiredness, malaise, and myalgias. After receiving CAR-T cells, the onset of fever can occur within a few hours to more than a week [21]. Patients often show the first CRS symptoms 14 days after receiving CAR-T cells; delayed CRS is rarer [22,23]. After the onset of fever, patients may develop various hematologic and organ toxicities, some of which may be life-threatening [24,25,26,27], with capillary leaks causing pulmonary and peripheral edema, multiorgan failure, hypotension, and circulatory system collapse.

Hematologic toxicities are also prevalent following CAR-T cell injection. Disseminated intravascular coagulation, prolonged prothrombin time (PT), partial thromboplastin time (PTT), and decreased fibrinogen have all been reported. Hemorrhagic events after treatment with CAR-T cells have resulted in patient deaths [26,28,29]. Moreover, following LDC, cytopenia is common, and delayed cytopenia is common after severe CRS [30]. Hemophagocytic lymph histiocytosis (HLH) has been observed during CRS [27], characterized with severe systemic hyperinflammation. Unremitting high fevers, cytopenia, coagulopathy, and an increase in typical HLH biomarkers are some of the defining characteristics of HLH.

Patients afflicted with CRS may experience wide-ranging organ dysfunction over time. Reports have indicated that myalgia is associated with increased creatine phosphokinase levels, which may suggest inflammatory muscle damage [25,31,32]. Furthermore, temporary increases in bilirubin and liver enzymes have been noticed [33,34], indicating hepatitis and liver failure [35]. Renal insufficiency may occur with a temporary rise in serum creatinine [15]. Hypotension, arrhythmias, and left ventricular systolic dysfunction are the most frequent cardiac problems that occur after CAR-T cell therapies in both adult and juvenile populations, and these conditions can result in overt heart failure. CRS may worsen such occurrences, leading to undesirable cardiovascular outcomes [36,37].

The administration of CAR-T cells with the B cell maturation antigen (BCMA) target was associated with neutropenia, thrombocytopenia, and anemia. The patients also developed a grade 1 gastrointestinal hemorrhage and grade 1~2 hypoproteinemia, and hematological damage [38]. Severe CRS cases can lead to a condition known as capillary leak syndrome (CLS). CLS involves fluid leakage from the bloodstream into surrounding tissues due to increased capillary permeability. This fluid shift can dilute the concentration of proteins in the blood, including albumin and other serum proteins, leading to hypoproteinemia, hypocalcemia, etc.

**Figure 3:**
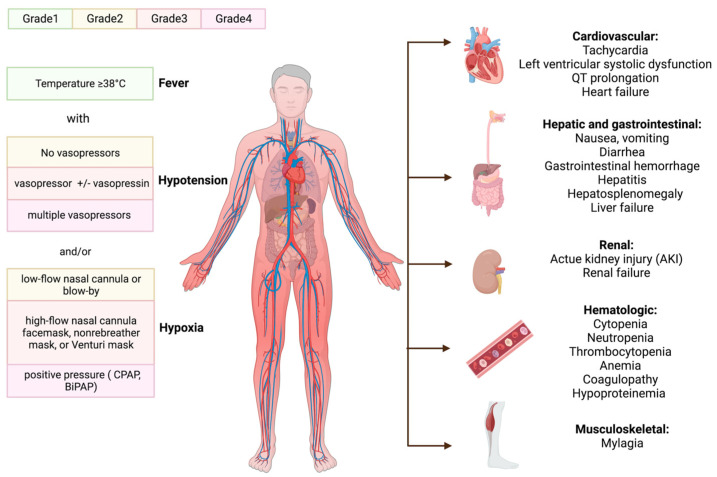
The manifestations of CRS and ASTCT CRS consensus grading. The initial fever may progress to numerous hematologic and organ toxicities before the clinical signs of cytokine release syndrome (CRS) appear. ASTCT classified it into four grades based on its chief manifestation: hypoxia, hypotension, and fever. Continuous positive pressure airway (CPAP); bilevel positive airway pressure (BiPAP).

The American Society for Transplantation and Cellular Therapy (ASTCT) summarized the consensus guidelines for grading CRS, with the severity of CRS depending on the level of temperature, hypotension, and hypoxia (Figure 3) [39].

### 2.2. Immune-Effector-Cell-Associated Neurotoxicity Syndrome (ICANS)

ICANS, formerly known as CAR-T-cell-related encephalopathy syndrome, is the second most frequent adverse impact of CAR-T cells. ICANS tends to be cytokine-mediated neurotoxicity rather than the OTOT impact, albeit the pathophysiology is still unknown. Several laboratory studies have proposed that endothelial activation contributes to ICANS pathogenesis [40]. CRS-associated endothelial activation can disrupt the blood–brain barrier (BBB) and infiltration of the cerebrospinal fluid with inflammatory cytokines and leukocytes. The central nervous system (CNS) can become inflamed when cytokines and immune cells reach the brain parenchyma, impairing or damaging neurons. It is anticipated that CAR-T cells, monocytes, and macrophages will be drawn to the CNS and release cytokines, which constitute vital elements of ICANS [41,42,43]. Mural cells, referred to as pericytes on the capillaries, are essential for maintaining the blood–brain barrier’s structure and encircle the endothelium, expressing CD19, which increases the BBB’s leakiness and causes ICANS in CD19-directed treatments [44].

**Figure 4:**
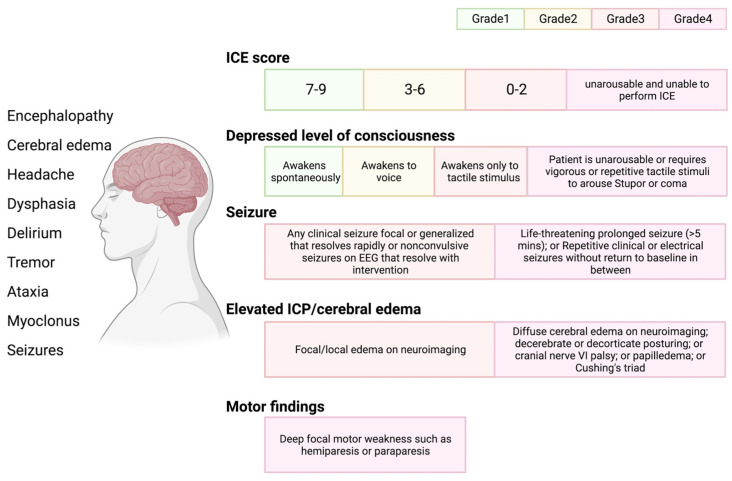
ICANS and ASTCT CRS consensus grading for adults. Immune effector cell associated neurotoxicity syndrome (ICANS) includes multiple distinct neurologic symptoms. ASTCT graded ICANS into four categories based on an immune effector cell associated encephalopathy (ICE) score: depressed level of consciousness, seizure, elevated intracranial pressure (ICP)/cerebral edema, and motor findings.

ICANS can be associated with various neurologic symptoms, yet these frequently evolve in a particular way. ICANS has been observed as early as the fourth or fifth day following CAR-T cell reinfusion and as late as the third or fourth week [45,46]. ICANS rarely develops in patients without prior CRS, but in these circumstances, it is often mild [23]. ICANS encompasses headache, tremors, impaired speech, confusion, delirium, poor consciousness (obtundation, lethargy, and stupor), and, less frequently, localized abnormalities [47]. The severity of ICANS is characterized and evaluated based on the American Society for Transplantation and Cellular Therapy (ASTCT) consensus criteria, which employ a method for assessing immunological functional cell-associated encephalopathy (Figure 4) [39].

### 2.3. Tumor Lysis Syndrome (TLS)

When a person experiences an effective cancer treatment (due to cell death), large amounts of phosphate, potassium, and nucleic acids can be released into their bloodstream, which can cause tumor lysis syndrome (TLS) [48]. While some cases of TLS have been attributed to chemotherapy, CAR-T cell infusion has also led to acute anaphylaxis and TLS in some cases, even without prior conditioning chemotherapy [27,49,50]. Indeed, rapid lymphoma cell death after CAR T cell treatment can be problematic if the kidneys cannot metabolize the byproducts of tumor cell lysis rapidly enough, resulting in hyperuricemia, hyperkalemia, hyperphosphatemia, and hypocalcemia. Acute kidney damage can also be exacerbated due to the accumulation of uric acid, phosphates of calcium, and ferritin, resulting in systemic inflammation and iron overload.

### 2.4. On-Target, Off-Tumor Toxicity (OTOT)

Ideally, only the malignant cells will express a CAR-T cell target antigen, leaving healthy tissue unaffected. Solid tumors have had only patchy success with CAR-T cell treatment. A great deal of malignant antigens are tumor-associated antigens (TAAs), which are expressed on both healthy and tumor tissues. As a result, CAR-T cells are frequently unable to distinguish normal cells from malignant cells, resulting in the assault and death of normal cells, known as “on-target, off-tumor” toxicity [51,52]. OTOT is more likely to occur in solid tumors; therefore, greater research efforts are required to identify tumor-specific antigens (TSAs). In fact, several cases highlight the difficulties associated with TAA expression on normal tissue. For example, the first patient to receive CAR-T cells targeting HER2 experienced respiratory distress and a sizeable pulmonary infiltrate within 15 min of cell infusion, ultimately leading to lung damage and death [10]. Lung toxicity was also observed in other clinical trials testing CAR-T cells directed against CEA [51].

### 2.5. Additional Factors Associated with Toxicity

It is crucial to note that the incidence of side effects in CAR-T cell therapy might vary greatly based on the specific CAR-T cell product, the kind of cancer, and individual patient variables. In vivo, CAR-T cell expansion and toxicity may be exacerbated with tumor load, degree of conditioning therapy, higher infusion dose, and CAR design. Indeed, juvenile B-ALL patients with a higher baseline tumor burden exhibit more considerable CAR-T cell proliferation and severer CRS [53,54]. Large tumor loads in patients are also associated with higher severity and incidence of the syndrome, likely due to the higher levels of T cell activation observed in clinical studies [34,55]. Patients with a greater ALL burden and those who underwent higher infusion dosages of CD19 CAR-T cells were shown to have a higher incidence of CRS [25,56,57].

CRS has been observed to begin earlier when CAR-T cells are constructed with CD28 costimulatory domains (as opposed to a 4-1BB costimulatory domain) [33]. The CAR hinge region binds the scFv to the transmembrane part of the protein, and deleting the flexible amino acid glycine in this area may reduce CAR-T cell over-activation [58]. The reduced flexibility in the CD8α hinge increased survival in preclinical investigations for high tumor burden and decreased proinflammatory cytokines, indicating potential benefits for safety and efficacy [59,60].

Enhancing the efficacy and duration of CAR-T cell responsiveness is the primary objective of conditioning therapy to improve the overall clinical outcomes in cancer patients. Although these negative consequences have also been recorded in the absence of chemotherapy conditioning, it is known that conditioned therapy is associated with the development of thrombocytopenia, anemia, and neutropenia [31,61,62].

## 3. Institutional Management Strategies for CAR-T Cell Toxicity

The life-threatening side effects of CAR-T cell treatment must be controlled with precisely forecasted and quick identification, as well as suitable actions to manage the toxicity in advance or prevent its deterioration. This is necessary to strike a balance between safety and efficacy.

### 3.1. Prediction and Prevention of Side Effects

Various research strategies have been developed to predict and prevent toxic adverse effects. Monitoring important indicators is recommended for all patients during the therapy, mainly focusing on cardiovascular, pulmonary, and neurologic systems. The association between the development of severe CRS and clinical indicators is imprecise, and the creation of predictive biomarkers for serious side effects is required. CRP, an indicator of inflammation, can aid in the early detection and management of CRS. Elevated LDH (an enzyme secreted when tissues/cells are destroyed) can indicate inflammation, tissue damage, or TLS. Cytopenia can result from CAR-T cell treatment. A reduction in red blood cells (anemia), white blood cells (leukopenia), and platelets (thrombocytopenia) may occur. Monitoring CBC levels assists healthcare providers in identifying and managing these concerns as soon as possible. A complete blood count with differential (CBC), C-reactive protein (CRP), lactate dehydrogenase (LDH), coagulation tests, uric acid, and ferritin should be included in the baseline laboratory evaluation [22,57], with repeat CRP and ferritin suggested with NCCN guidelines three times per week for 2 weeks after infusion [63].

According to several studies, people with severe CRS had higher serum GM-CSF levels [15]. In addition, it has been shown that activated CAR-T cells enhance GM-CSF receptor expression [64], suggesting that GM-CSF might be crucial in the emergence of CRS. Based on these observations, we can predict the occurrence and development of diseases in advance and control them in time. To diagnose patients with undetected cardiovascular disease, baseline medical history, a physical examination, an electrocardiogram (ECG), an echocardiogram, and cardiac biomarkers should be acquired [64]. Moreover, early detection of tumor lysis can lessen the risk of fatal cardiac arrhythmias, convulsions, and other complications [48].

In addition to thorough conventional clinical observations, laboratory tests, and biomarkers, more efficient predictors for early intervention and therapy are urgently needed. Studies have discovered that patients with severe CRS have higher serum levels of angiopoietin (Ang)-1, Ang-2, sE-selectin, and soluble intercellular adhesion molecule (sICAM)-1 [65]. These factors indicate that the pathological activation of endothelial cells affects the seriousness and development of the disease. Moreover, CRS severity assessments might be possible using soluble vascular cell adhesion molecule (sVCAM)-1 [66]. Designing CAR-T cells that can differentiate between cancer cells and cancer-free cells is essential to lower the risk of harm, apart from looking for antigens with limited expression on normal cells like folate receptor 1 (FOLR1) and tumor-associated glycoprotein 72 (TAG72) [67,68].

First-generation CARs are made up of an antigen-binding domain connected to a CD3ζ. Second-generation CARs were improved by integrating an additional co-stimulatory signaling domain, such as CD28 or 4-1BB. Third-generation CARs built on the second-generation by incorporating two or more co-stimulatory domains, typically a mix of CD28 and 4-1BB or other co-stimulatory molecules. CARs of the fourth generation integrate CAR technology with cytokine expression systems [69]. However, in addition to better efficacy, some later generations are associated with an increased risk of severe side effects. Ongoing research aims to improve patient outcomes by optimizing CAR-T cell therapies to establish a balance between safety and effectiveness.

‘IF/THEN’ [52], ‘AND’ [70], ‘OR’ [71], and ‘NOT’ [72] logic gates could be implemented in controlling the activation of CAR-T cells. The SynNotch technology includes a synthetic receptor called the “SynNotch receptor”, which allows modified T cells to identify a specific antigen on the tumor cell surface. Traditional CAR T cells may accidentally assault healthy tissues presenting low quantities of the targeted antigen, resulting in toxicities and undesirable side effects. SynNotch-CAR T cells, with their sequential identification process, dramatically reduce the likelihood of severe toxicities [73,74,75].

Regional or local administration in solid tumors could help to minimize trafficking to non-malignant tissues, including intracranial transport [76,77,78], intra-tumoral injection [79], intrapleural injection [80], and intra-arterial hepatic infusion [81]. Lowering the danger of injury requires designing CAR-T cells that can distinguish cancerous and normal cells [82]. Pre-infusion bridging strategies such as traditional chemotherapy and radiotherapy have shown efficacy in reducing tumor burden ahead of CAR-T cell therapy, minimizing the potential of tumor lysis and the severer CRS symptom [83].

As the early and aggressive use of immunosuppressive therapy could potentially hinder the efficacy of immunotherapy, current clinical strategies aim to minimize its use in patients at risk of experiencing severe and possibly life-threatening consequences of cytokine release syndrome.

### 3.2. Treatment and Supportive Care

When adverse reactions occur, prompt and effective treatment measures must be taken (Table 1). Supportive treatment and close surveillance are the primary management strategies for grade 1 CRS and ICANS.

Corticosteroids account for a large portion of CAR-T cell toxicity control because they directly impact CAR-T cell proliferation and function. They may also hamper the production of several other cytokines and chemokines, causing systemic immunological suppression [45]. High-dose corticosteroids can occasionally minimize toxicity, yet they can also decrease T cells’ proliferation, function, and activation, which can diminish the CAR T cell therapy’s clinical effectiveness [15,57]. A study has found that early steroid administration may prevent serious ICANS without reducing CAR T cell effectiveness [86].

In contrast with mild CRS, which can be treated with supportive care alone, severe CRS is typically treated with tocilizumab. This recombinant humanized monoclonal antibody is directed against the interleukin-6 receptor (IL-6R). The fundamental idea behind cytokine-based therapy is IL6-directed therapy. In 2017, the U.S. Food and Drug Administration approved tocilizumab to manage severe or life-threatening CAR-T-cell-induced CRS. This was based on a retrospective examination of data from clinical studies [87]. Limited corticosteroid therapy is, in some cases, utilized to manage CRS further [26,29]. Siltuximab is the new anti-IL-6 chimeric monoclonal antibody [88]. In contrast to tocilizumab, siltuximab may have the advantage of avoiding ICANS by eliminating IL-6 from circulation. Tocilizumab does not impact the efficacy or expansion of CAR-T cells, in contrast to corticosteroids [89]. Of particular significance, an analysis of 60 patients with CAR-T-cell-linked CRS shows that tocilizumab exhibits outstanding effectiveness in managing severe CRS. No adverse events related to the administration of tocilizumab were found [87].

Significantly, tocilizumab fails to cross the blood–brain barrier, which means neurotoxicity is not addressed. Patients who suffer from neurotoxicity after receiving CAR-T cell therapy need simultaneous corticosteroid administration. There is no proven prophylactic method to stop ICANS. However, strategies to lessen CRS might subsequently restrict the risk of ICANS. GM-CSF deficiency or inhibition has been demonstrated to alleviate CRS and ICANS, increasing the anti-tumor effects of CAR-T cell therapies by deterring the local invasion of myeloid cells and T cells [90,91].

Recent studies revealed that anakinra could mitigate ICANS [92,93]. In a single-center experiment, patients with steroid-refractory ICANS were treated with anakinra after standard CD19-targeting CAR-T cell treatment, and no significant effect on neurotoxicity was observed [94]. These studies indicated that anakinra may be an effective treatment of steroid-refractory or progressive ICANs caused by CAR-T cell therapy.

Symptomatic and supportive treatments, including antipyretics for fever, vasopressor support, mechanical ventilation in cases of respiratory failure, temporary hemodialysis support in severe instances, and intravenous hydration to maintain urine flow, are recommended to manage adverse events associated with CAR-T cell therapy. Clinical examinations are necessary for a specific assessment and follow up throughout CAR-T cell treatment. The inclusion of treatment-related examinations in patient monitoring protocols can enable the timely evaluation of changes in patient indices before and after treatment (Table 2). The attending physician determines the appropriate test items and frequency for cases with rapidly changing conditions.

Categorizing toxicity and side effects through this approach is valuable when selecting the appropriate targeted treatment strategies to guide therapeutic interventions in clinical trials. Underlining the importance of prompt identification and management of CAR-T cell toxicity may greatly enhance results and lessen the burden brought on with associated complications. In addition, these tests could confirm whether early action will reduce toxicity without impacting efficacy [95].

## 4. Conclusions

CAR-T cell therapy is an innovative and promising treatment that has demonstrated the potential to expand its application on relapsed/refractory hematological malignancies or solid tumors. However, CAR-T cell treatment’s therapeutic benefits come with the possibility of harmful side effects. Monitoring important indicators and related clinical examination could be applied for early intervention signs and therapy effectiveness evaluation. An optimized structure, logic gates, specific antigens, and the regional delivery of CAR-T cells have demonstrated efficacy and potential in minimizing OTOT. The consensus guidelines, ASTCT grading, could be used widely in both ordinary medical practice and clinical trials, which can contribute to standardizing the management of grade-dependent CRS and ICANS. It is crucial to guard against and manage these toxic effects based on an in-depth comprehension of pathogenesis and clinical manifestations, and future research should involve efforts to assess CAR-T cell toxicities to optimize the safety and efficacy of this treatment.

## Figures and Tables

**Table 1:** The management of CRS and ICANS with different grades.

Side Effects		Grade 1	Grade 2	Grade 3	Grade 4
CRS [84,85]	Fever or organ toxicity	Acetaminophen or Ibuprofen can be used as a treatment option for fever Assess for infection using blood and urine cultures, and chest radiography Empiric broad-spectrum antibiotics and filgrastim if neutropenic Maintenance of intravenous (i.v.) fluids for hydration Symptomatic management of constitutional symptoms and organ toxicities Tocilizumab or siltuximab ± corticosteroids			
	Hypotension	Grading and supportive care	Echocardiogram; initiate other methods of hemodynamic monitoring i.v. fluid bolus of 500–1000 mL of normal saline Tocilizumab or siltuximab; tocilizumab can be repeated after 6 h if needed		
			If hypotension persists after two fluid boluses and anti-IL-6 therapy, start vasopressors, and consider transfer to an intensive care unit	Monitoring in the intensive care unit	
			In patients at high risk or hypotension persists after 1–2 doses of anti-IL-6 therapy, dexamethasone can be used at 10 mg i.v. every 6 h for 1–3 days	Dexamethasone at 10 mg i.v. every 6 h for 1–3 days If refractory, increase to 20 mg i.v. every 6 h	Methylprednisolone i.v. 1000 mg/day for 3 days 250 mg × 2/day for 2 days 125 mg × 2/day for 2 days 60 mg × 2/day for 2 days
	Hypoxia	Grading and supportive care	Tocilizumab or siltuximab ± corticosteroids and supportive care		
			Supplemental oxygen	Supplemental oxygen, including high-flow oxygen delivery and non-invasive positive-pressure ventilation	Mechanical ventilation
ICANS [45,85]		Supportive care and neurological work-up: Aspiration precautions and i.v. hydration Seizure prophylaxis with levetiracetam EEG Brain imaging (MRI and CT) Spinal imaging for focal motor weakness Consider tocilizumab if there is concurrent CRS			
			Transferring the patient to an intensive care unit if grade ≥ 2 CRS	Intensive care unit transfer	Intensive care unit monitoring; mechanical ventilation
			Dexamethasone at 10–20 mg i.v. every 6 h or its equivalent of methylprednisolone for 1–3 days.		Lower ICP with hyperventilation, hyperosmolar therapy with mannitol/hypertonic saline, and/or neurosurgery consultation for a ventriculoperitoneal shunt in patients with cerebral edema
				Control seizures with benzodiazepines (for short-term control) and levetiracetam +/− phenobarbital and/or lacosamide For focal/local edema, methylprednisolone i.v. 1000 mg/day for 3 days 250 mg × 2/day for 2 days 125 mg × 2/day for 2 days 60 mg × 2/day for 2 days	

CRS: cytokine release syndrome; EEG: electroencephalogram; ICANs: immune-effector-cell-associated neurotoxicity syndrome; ICP: intracranial pressure; i.v.: intravenous injection.

**Table 2:** CAR-T cell therapy related examination for management of toxicities.

Classification of Examination	Specific Examination and Tests
Regular laboratory examination	Blood routine; blood biochemistry; coagulation function test; arterial blood gas analysis; infection-related test; cytokines (IL-1, IL-2, IL-6, TNF-α, IFN-γ, etc.)
Regular imaging examination	Chest and abdomen enhanced CT; cerebral enhanced MRI; abdominal ultrasound; echocardiogram
Proliferation of CAR-T cells in vivo	Quantitative PCR detection of peripheral blood CAR gene; flow cytometry
Examination of organ function	Electrocardiogram; echocardiogram; systemic, superficial lymph node ultrasound; lung function test; CARTOX-10 scoring; electroencephalogram; cerebrospinal fluid pressure; abdominal ultrasound; (regular laboratory examination is helpful to assess organ function)

CAR-T-cell therapy associated toxicity-10 (CARTOX-10).

## Data Availability

The original contributions presented in the study are included in the article files; further inquiries can be directed to the corresponding authors.

## List of Abbreviations

SCV	single chain of variable fragment
LDC	lymphodepletion chemotherapy
CRS	cytokine release syndrome
ICANS	immune-effector-cell-associated neurotoxicity syndrome
IFN-γ	interferon-gamma
IL-6	interleukin-6
TNF-α	tumor necrosis factor-alpha
GM-CSF	granulocyte–macrophage colony-stimulating factor
APCs	antigen-presenting cells
DCs	dendritic cells
CD40L	ligand for CD40
DAMPs	danger-associated molecular patterns
PT	prothrombin time
PPT	partial thromboplastin time
HLH	hemophagocytic lymph histiocytosis
ASTCT	American Society for Transplantation and Cellular Therapy
BBB	blood–brain barrier
CNS	central nervous system
TLS	tumor lysis syndrome
BCMA	B cell maturation antigen
CLS	capillary leak syndrome
OTOT	on-target, off-tumor toxicity
TAAs	tumor-associated antigens
TSAs	tumor-specific antigens
CBC	complete blood count
CRP	C-reactive protein
LDH	lactate dehydrogenase
ECG	electrocardiogram
sICAM	soluble intercellular adhesion molecule
sVCAM	soluble vascular cell adhesion molecule
FOLR1	folate receptor 1
TAG72	tumor-associated glycoprotein 72
IL-6R	interleukin-6 receptor
i.v.	intravenous injection
ICP	intracranial pressure
